# Delta Event-Related Oscillations Are Related to a History of Extreme Binge Drinking in Adolescence and Lifetime Suicide Risk

**DOI:** 10.3390/bs10100154

**Published:** 2020-10-07

**Authors:** Cindy L. Ehlers, Derek N. Wills, Katherine J. Karriker-Jaffe, David A. Gilder, Evelyn Phillips, Rebecca A. Bernert

**Affiliations:** 1 Department of Neuroscience, The Scripps Research Institute, 10550 North Torrey Pines Road, La Jolla, CA 92037, USA; dnwills@scripps.edu (D.N.W.); dgilder@scripps.edu (D.A.G.); ephillip@scripps.edu (E.P.); 2 RTI International, Research Triangle Park, NC 27709, USA; kkarrikerjaffe@rti.org; 3 Department of Psychiatry and Behavioral Sciences, Stanford University, Stanford, CA 94305, USA; rbernert@stanford.edu

**Keywords:** adolescence, binge drinking, suicidal behaviors

## Abstract

Alcohol exposure typically begins in adolescence, and heavy binge drinking is associated with health risk behaviors. Event-related oscillations (EROs) may represent sensitive biomarkers or endophenotypes for early alcohol exposure as well as other risk behaviors such as suicidal thoughts and actions. In this study, young adults (age 18–30 years) of American Indian (AI) (*n* = 479) and Mexican American (MA) (*n* = 705) ancestry were clinically assessed, and EROs were generated to happy, sad and neutral faces. Extreme adolescent binge drinking (10+ drinks) was common (20%) in this population of AI/MA and associated with a significantly increased risk of a lifetime history of suicidal acts (SA, suicide attempts, deaths) but not suicidal thoughts (ST, ideation, plans). ST were reported among MA participants, whereas SA were more common among AI young adults. Extreme adolescent binge drinking was also associated with errors in detection of sad and neutral faces, increases in delta ERO energy, and decreases in phase locking (PL), particularly in parietal areas. A lifetime history of ST was associated with increases in delta ERO energy and PL, whereas SA were associated with decreases in both. These studies suggest that ERO measures may represent important potential biomarkers of adolescent extreme binge drinking and risk for suicidal behaviors.

## 1. Introduction

Alcohol use disorders (AUD) and problems usually follow a developmental trajectory that can begin in early adolescence, continuing into young adulthood, and persisting into older ages [1,2,3,4]. When adolescents and young adults begin to drink, they often engage in “binge drinking” (5 drinks for boys, 4 for girls per occasion) [5,6]. There are emerging data demonstrating that some adolescents report drinking amounts that are double or more the standard binge threshold, consuming 10+ or 15+ drinks per occasion, which has been called “high-intensity drinking” or “extreme binge drinking” [6,7,8,9]. Heavy and frequent binge drinking during adolescence has been linked to increased risk for AUD in young adulthood [10] (Ehlers 2020, in submission) as well as other health risk behaviors [11] including suicide [12,13,14].

The evaluation of early extreme binge drinking in specific ethnic groups, such as American Indians (AI), may be of particular importance because several studies have found that AI adolescents report the highest rate of past-month binge drinking when compared to White and mixed-race adolescents in the U.S. [15,16,17]. Understanding binge drinking in Hispanic American subgroups, such as Mexican Americans (MA), is also important because they represent the largest subgroup of Hispanic Americans and have the highest prevalence of heavy drinking and alcohol use disorders among Hispanics [18,19,20]. 

Identifying AI and MA adolescents engaging in heavy binge drinking may aid in the development of early intervention and prevention programs for substance use disorders as well as associated health risks, including suicide. Suicide is a preventable public health problem with a global disease burden that ranks as the second leading cause of death among young adults in the US (aged 15–29) [21]. According to the Institute of Medicine, suicidal behaviors are prevalent, with approximately 25 suicide attempts (100–200 for youth) estimated to occur for every death by suicide [22]. Suicide rates are alarmingly high among American Indians/Alaska Natives (AI/AN), with rates 50% higher compared to White persons across all age groups. Among AI/AN young people, suicide has emerged as a public health emergency, with rates that are 3 times higher than the US rate for all races combined [23,24,25]. Alcohol use disorders confer known risk for suicidal behaviors, with recent use among AI/AN associated with twice the number of suicide deaths [26]. Although Mexican Americans show lower rates of suicide relative to other ethnic groups [27], alcohol use represents a critical modifiable risk factor prioritized for early intervention and targeted for increased research to inform suicide prevention [28,29,30,31].

Investigation of underlying biomarkers of risk may be helpful in understanding the impact of adolescent extreme binge drinking and concomitant negative health behaviors. Electrophysiological variables represent some of the most reliable and sensitive biomarkers of vulnerability to—or endophenotypes for—alcohol-related psychopathology (for extensive reviews see: [32,33,34,35,36,37,38,39,40]). Reduced amplitude of the P300 or P3 component of the event-related potential (ERP) has perhaps received the most attention as a possible biomarker for AUD risk [41,42,43,44,45,46,47,48,49,50,51,52,53,54]. Several studies have recently applied time-frequency techniques, such as event-related oscillations (EROs), to ERP data in order to develop additional measures of alcohol-related psychopathology [36,38,55,56,57,58,59]. The application of ERO analyses can generate information on the amount of energy in different frequency sub-bands (such as delta, theta, alpha, and beta activity) that may contribute to the ERP waveform. Using ERO techniques, Basar and colleagues were the first to demonstrate that the P300 wave is composed of an amplitude enhancement of delta, theta, and alpha frequency components of the pre-stimulus EEG (see [60,61,62]). Additionally, it has been suggested that the main portion of P300 power is most likely within sub-delta and delta bands, especially since the P300 is virtually abolished with a high-pass setting at 1.0 Hz [63] or 2.0 Hz [64].

ERO energy measures, in different frequency sub-bands, have also been applied to the investigation of alcohol-related psychopathology [36,38,39,55,56,57,58,59,65]. For instance, it has been demonstrated that both alcoholics [66] and young offspring of alcoholics, who are at high risk for alcohol use disorders [67], show decreases in delta oscillations in response to target stimuli during a visual oddball task. Genetic mouse models of high alcohol preference also have demonstrated that delta oscillatory responses are less robust in alcohol-preferring mice as compared to non-preferring mice using an auditory oddball paradigm [68]. While a number of paradigms have been used to generate delta oscillatory responses, it has been suggested that delta responses, particularly in temporal-parietal-occipital locations in humans, are highly sensitive to the perception of facial expressions [69,70,71]. In fact, it has been proposed that increases in delta responses in occipital regions could be specific to the perception of faces and facial expressions [72]. Thus, evaluating posterior delta oscillatory responses using a facial recognition paradigm may provide important emotion-based measures through which to assess alcohol pathology. 

Other measures that can be extracted from ERP data using time-frequency analyses, which are also relevant to alcohol pathology, are neuronal synchrony measures. These measures quantify the degree to which groups of neuronal ensembles begin oscillating within a specific frequency range and then enter into phase locking, or synchrony, with each other over a specified period of time [73,74,75]. Neural phase locking can be measured over time within a local neuronal population (‘local synchrony’) or between distant populations (‘long-range synchrony’) [76,77], and thus represents a process whereby communication can effectively occur either within a brain area or between brain areas. ERO synchrony measures have been demonstrated to be a sensitive index of brain development over the course of adolescence in humans and rodents [78] as well as a potential measure of risk for AUD [57,59]. Thus, measures of neuronal energy and phase synchrony, within specific frequency bands, may also represent possible biomarkers of adolescent extreme binge drinking as well as concomitant suicide risk. 

The present analysis is part of a larger study exploring risk factors for substance dependence among American Indians and Mexican Americans residing in Southwest California [79,80,81,82,83,84,85,86,87,88,89,90,91,92,93]. The purpose of the present work was to use a facial discrimination task developed by Erwin et al [94], which we have adapted to a visual ERP paradigm, to study oscillatory responses in the delta frequency range in a young adult population of AI and MA. An affective-target paradigm was employed; this has been used in our previous studies to investigate ERP responses to affective stimuli (see [45,95,96,97,98,99]). The study had two specific aims: (1) to evaluate ERO amplitude and synchrony, in the delta frequency range, to facial expressions (happy, sad, and neutral) in a population of AI and MA young adults, and (2) to determine if ERO differences in the delta frequency range are associated with an adolescent history of extreme binge drinking and suicidal thoughts and behaviors. While a number of studies have commented and documented the prevalence of both binge drinking and suicidal behaviors in AI, this is the first study to attempt to identify biomarkers of these phenomena in AI and MA participants. 

## 2. Materials and Methods

### 2.1. Participants

The participants were individuals of Mexican American (MA) and American Indian (AI) ancestry that were recruited from the local community for studies investigating risk factors for substance dependence in these two ethnic groups. American Indian participants were recruited from eight geographically contiguous reservations with a total population of about 4800 individuals. They were recruited using a combination of a venue-based method and a respondent-driven procedure from May 1995 to December 2019, as described previously [79]. American Indian participants were recruited in a wide age range (18–82 years); however, the present set of analyses focuses only on those aged 18–30 years at the time of interview. Mexican American participants were recruited using a commercial mailing list that provided addresses of individuals with Hispanic surnames in 11 ZIP codes in San Diego County, each of which had a population that was over 20% Hispanic and was within 25 miles of the research site. The mailed invitation stated that potential participants must be of Mexican American ancestry, be between the ages of 18 and 30 years, be residing in the United States legally, and be able to read and write in English. They were recruited between October 2003 to December 2019 (see [92]). Based on the aims of the larger studies for both ethnic groups, participants were excluded if they were pregnant, breastfeeding, or currently had a major medical disorder that precluded their traveling to the research site. Participants were asked to refrain from alcohol or any other substance use for 24 h prior to testing, and their breathalyzer blood alcohol levels had to be 0.00 g/dl to be included in the study.

Potential participants gave written informed consent and then responded to a screening questionnaire that was used to gather information on demographics, personal medical history, ethnicity, and detailed measures of current and past substance abuse history, including a retrospective report of their adolescent binge drinking using a timeline follow-back format. An adolescent history of extreme binge drinking was defined as drinking 10 or more drinks per drinking occasion at least once a month during their heaviest drinking period at or before the age of 19 years. Each participant also completed a Semi-Structured Assessment for the Genetics of Alcoholism (SSAGA) interview, which collected information including demographics, psychiatric history, lifetime history of lifetime suicidal behaviors (suicidal thoughts (ST): suicidal ideation and plans and/or suicidal acts (SA): suicide attempts. Information on suicide deaths was obtained from community sources (e.g., verified by public records, family informants, etc.) [100]. 

### 2.2. ERP Recordings

Seven channels of ERP data (FZ, CZ, PZ, F3, F4, F7, and F8); referenced to linked ear lobes with a forehead ground; international 10–20 system) were obtained using gold-plated electrodes with impedances held below 5 KΩ. Frontal electrodes were emphasized in the montage, as previous data suggested that P3 decrements in frontal and parietal areas distinguished subjects at risk of alcohol dependence (see [101,102]). An electrode placed left lateral, infraorbitally, and referenced to the left earlobe was used to monitor both horizontal and vertical eye movements. ERP signals were amplified (time constant 0.3 s, 35 Hz low pass) using a Nihon Kohden EEG instrument, and were transferred on-line to a digital multiplexer amplifier (50 K). The stimuli were realistic digital photographs of happy, neutral, and sad faces presented on a computer screen for 1000 ms with an inter-trial interval of 1000–1500 ms. The pre-stimulus interval was 150 ms. Participants were instructed to depress one counter whenever a happy and another whenever a sad face was displayed (36 trials each) and not to respond to neutral faces (144 trials). There were 36 total faces (12 each of happy, neutral, and sad) presented in a random order for a total of 216 trials. The number of male and female faces presented was also equally distributed among neutral, sad, and happy stimuli. The percent of the stimuli that was correctly identified was calculated for each participant.

### 2.3. ERO Analyses

The ERO trials were digitized at a rate of 256 Hz. Trials containing excessive artifact were eliminated prior to averaging (<5% of the trials). An artifact rejection program was utilized to eliminate individual trials in which the EEG exceeded ± 400 µV. Data from single trials generated by the stimuli were entered into the time-frequency analysis algorithm. The *S*-transform (ST), a generalization of the Gabor transform [103], was used (see [104]). The *S*-transform results in a time-frequency representation of the data. The exact code we used is a C language, *S*-transform subroutine available from the NIMH MEG Core Facility web site Available online: https://kurage.nimh.nih.gov/meglab/Meg/Software (accessed on 5 October 2020). This code is specifically for use with real time series, so it sets the input imaginary values, required by the *S*-transform, to zero, and it always uses the Hilbert transform, so that each of the complex output time series is an analytic signal. To quantify *S*-transform magnitudes, a region of interest (ROI) was identified by specifying the band of frequencies and the time interval contained in the rectangular ROI. The time-frequency points saved from each *S*-transformation are from 100 ms before to 900 ms after the onset of the stimulus and from 1 Hz through 50 Hz at intervals of 0.5 Hz. Energy is the square of the magnitude of the *S*-transform output in a time-frequency region of interest. The *S*-transform output for a time/frequency ROI for a specific EEG lead is proportional to the input voltage of the lead over the time/frequency interval. The *S*-transform magnitude squared for a time/frequency interval is therefore proportional to volts squared. These analyses are similar to those which have been previously described [58]. The phase lock index (PLI) is a measure of synchrony of phase angle over multiple trials, as a function of the frequency and of the time relative to the start of the stimulus for each trial. The range of PLI is from zero to 1.0, with high values at a given time and frequency indicating little variation among trials of the phase angle at that specific time and frequency. The approach used combined event related oscillations (time locked to the stimuli, and both phase-locked and non-phase-locked to the stimuli onsets) into a single class of events, with the contribution to the P300 response depending on the degree to which the phase locking phenomena acted on this class of events. As an alternative, phase-locked and non-phase-locked EROs could have been conceptualized and measured as two separate classes of events, as has been done by others see [55,65]. 

**Figure 1:**
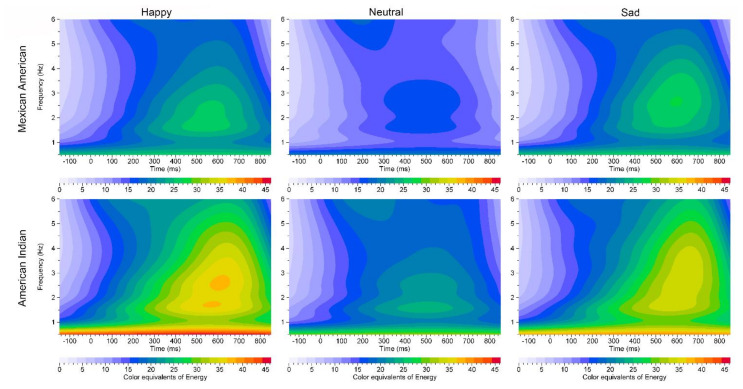
Event related oscillation (ERO) energy color equivalent maps in American Indians and Mexican Americans for each stimulus type. Parietal lead (PZ) electrode location is shown for the 0–6 Hz frequency range and −100 ms to 850 ms. Delta frequency band region of interest is (1–4 Hz) for the 200 to 500 ms time interval. Stimulus is presented at 0 ms.

Rectangular regions of interest (ROIs) were defined within the time-frequency analysis plane by specifying, for each ROI, a band of frequencies and a time interval relative to the stimulus onset time. The ROI frequencies tested were in the delta (1–4 Hz) frequency range and the ROI time interval was 200–500 ms, ROI time intervals were selected based on ERO energy in specific P3 ERP [78,105]. Using mean values over trials, the maximum energy values were calculated for each ROI at each electrode location. Representative ERO color maps of the PZ electrode location are shown in Figure 1 for both the American Indian and Mexican American subjects for all three stimulus types. 

### 2.4. Data Analyses

For the clinical comparisons, we first determined if histories of monthly extreme-binge drinking during adolescence and suicidal acts and behaviors were significantly associated with gender, socioeconomic status, education, race, and age. To determine associations between binge drinking levels and these covariates, we used analyses of variance (ANOVA) for continuous variables and Chi-square tests for dichotomous variables. Second, we assessed whether a history of adolescent extreme binge drinking was correlated with lifetime diagnosis of suicidal ideation or acts using G-tests that calculate the likelihood-ratio or maximum likelihood statistical significance using a log linear model. This a test that is increasingly being used in situations where Chi-squared tests were previously recommended [106]. Two 2 × 2 × 2 analyses that took into consideration a history of adolescent extreme binge drinking (vs. no binge drinking), race (AI vs. MA), and suicidal behaviors (1st analysis: Suicidal acts vs. no acts or thoughts; 2nd analysis: Suicidal thoughts vs. no acts or thoughts) were conducted. Third, for the ERO analyses, energy and synchrony in the delta frequency range at the three electrode locations (FZ, CZ, and PZ) were first compared, using repeated measures ANOVA, in all participants for the three facial expression categories (happy, sad, and neutral). Then, we compared ERO delta frequency energy and synchrony for all the demographic variables, using ANOVA. Finally, we compared responses for the three diagnostic categories: Extreme binge drinking vs. no extreme binge drinking; suicidal thoughts (ideation/plans) vs. no suicidal thoughts or acts; and suicidal behaviors (suicide attempts/deaths) vs. no suicidal behaviors or thoughts. For these analyses, participants’ demographics were included if they significantly impacted ERO variables in bivariate analyses. Significance for ERO analyses was set at *p* < 0.05. Analyses were conducted in SPSS [107].

## 3. Results

Clinical findings: The sample consisted of American Indian (*n* = 479) and Mexican American (*n* = 705) young adult (18–30 years old) participants. As shown in Table 1, 57.2% of the sample was female (AI:53.9%, MA:59.4%), 50.0% of the sample was currently employed (AI:30.8%, MA:62.7%), and 28.1% (AI:40.1%, MA:20.3%), of the sample had an annual income below $20,000. The mean age was 22.8 ± 3.92(SD) years (AI:21.5 ± 3.7, MA:23.7 ± 3.8), and the mean level of education was 12.7 ± 1.86(SD) years (AI:11.5 ± 1.3, MA:13.5 ± 1.8). There were 235 (AI:180, MA:55) participants who reported extreme binge drinking (>10 drinks per occasion), and the mean number of drinks they reported per occasion was 17.5 ± 10.0(SD) (AI:18.0 ± 10.3, MA:15.8 ± 9.0). 

**Figure 2:**
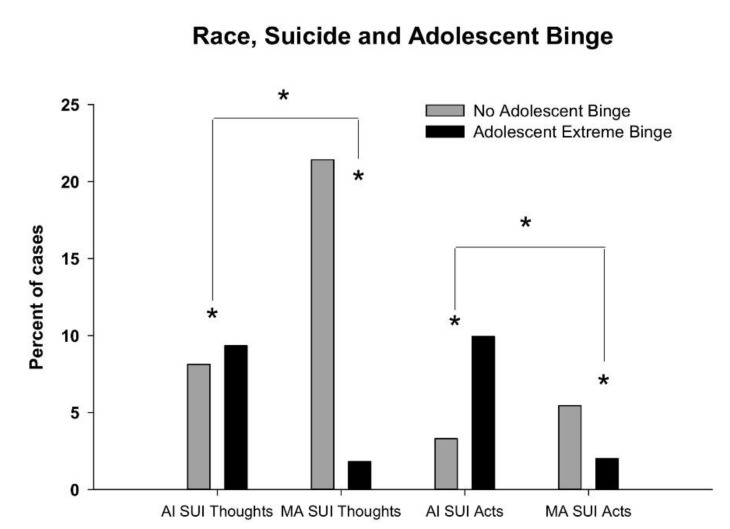
Suicide activity in American Indian (AI) and Mexican American (MA) participants with and without a history of adolescent extreme binge drinking. Percentage of subjects in each binge group shown for suicide activity: Thoughts or acts. Two 2 × 2 × 2 analyses were run comparing no suicide activity to thoughts in one, and acts in the second. Race and binge were included in both. * indicates G square *p*-value less than 0.001.

What is also shown in Table 1 is that participants with a history of adolescent extreme binge drinking differed on age (younger, F = 9.98, df = 1, *p* < 0.01), education level (fewer years education, F = 131.4, df = 1, *p* < 0.001), race (American Indian, Chi-squared = 181.5, df = 1, *p* < 0.001), gender (male, Chi Square= 16.4, df = 1, *p* < 0.001), employment (unemployed, Chi-squared = 34.1, df = 1, *p* < 0.001), economic status (income < $20,000, Chi-squared = 21.2, df = 1, *p* < 0.001), and marital status (unmarried, Chi-squared = 6.1, df = 1, *p* = 0.016), compared to participants without a history of adolescent extreme binge drinking. In this sample, 263 participants reported a history of suicidal thoughts (ideation/plans) and suicidal acts were observed among 122 participants (suicide attempt history, suicide death). Suicidal thoughts were significantly more common in MA (Chi-squared = 6.1, *p* < 0.01), and suicidal acts were more common in AI (Chi-squared = 4.6, *p* < 0.03), as well as among those with less education (F = 11.6, *p* < 0.001) and in women (Chi-squared = 7.35, *p* < 0.007). Four men and two women died by suicide, and all were AI. A 2 × 2 × 2 analysis comparing a history of adolescent binge drinking, race, and suicidal thoughts was overall significant (G square = 160.9, *p* < 0.0001) with post-hoc analyses showing that race by adolescent binge (G square = 157.5, *p* < 0.0001) but not race by suicidal thought or adolescent binge by suicidal thoughts were significant, as shown in Figure 2. As also shown in Figure 2, a 2 × 2 × 2 analysis comparing a history of adolescent binge drinking, race, and suicidal acts was also significant overall (G square = 160.9, *p* < 0.0001) with post-hoc analyses showing that race by adolescent binge (G square 138.8, *p* < 0.0001), race by suicidal thoughts (G square = 5.78, *p* < 0.02), and adolescent binge by suicidal acts were also all significant (G square = 22.02, *p* < 0.0001).

## ERO analyses

**Figure 3:**
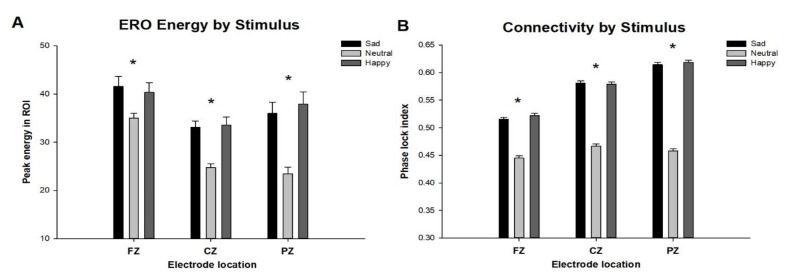
Event related oscillations (ERO) energy (**A**) and connectivity (**B**) comparing stimuli (Sad, Neutral, and Happy faces) in the delta frequency band (1–4 Hz) for the 200 to 500 ms time interval in all three electrode locations. Sad and Happy faces had significantly different energy and connectivity when compared to Neutral faces in all three electrode locations. * indicates repeated measure ANOVA *p*-value less than 0.01.

In all participants, ERO energy and connectivity (local phase locking) in the delta frequency range (1–4 Hz) was compared using repeated measures ANOVA, contrasting the target stimuli and the non-target stimuli for all three midline electrode locations. As seen in Figure 3A, ERO delta energy in responses to sad (FZ: F = 15.9, *p* < 0.0001; CZ: F = 95.3, *p* < 0.00001; PZ: F = 69, *p* < 0.0001) or happy (F = 13.1, *p* < 0.001; CZ: F = 53.9, *p* < 0.0001; PZ: F = 67.7, *p* < 0.0001) faces were significantly increased as compared to neutral faces, with significances being the greatest in the posterior leads. No differences in ERO delta energy were found when the trials between happy and sad faces were compared. 

As seen in Figure 3B, ERO connectivity in the delta frequency range in trials of sad (FZ: F = 276.4, *p* < 0.0001; CZ: F = 783.7, *p* < 0.00001; PZ: F = 1546.8, *p* < 0.0001) or happy (F = 360.8, *p* < 0.001; CZ: F = 847.9, *p* < 0.0001; PZ: F = 1749.1, *p* < 0.0001) faces was significantly increased as compared to trials of neutral faces with, once again, the differences being greatest in posterior leads. No differences in ERO delta connectivity were found when the trials between happy and sad faces were compared. ERO energy in the delta frequency range was compared for all demographic variables and was found to differ by age and race, whereas phase locking was found to differ by age and gender; these characteristics were then used as covariates in all subsequent ERO analyses. 

**Figure 4:**
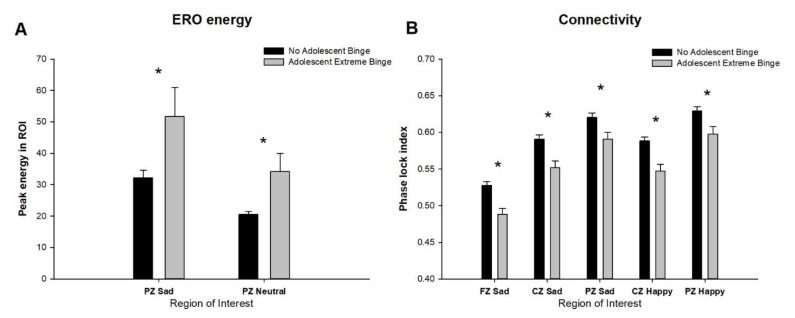
Event related oscillations (ERO) energy (**A**) and connectivity (**B**), shown in the delta frequency band (1–4 Hz) for the 200 to 500 ms time interval. Significant regions of interests (channel and stimulus type) are shown for subjects with and without a history of adolescent extreme binging. * indicates ANOVA co-varied for age and race *p*-value less than 0.05.

Adolescent extreme binge drinking also was associated with changes in ERO energy and connectivity, as seen in Figure 4. An increase in delta ERO energy in those with a history of adolescent extreme binge drinking was found in the parietal lead (PZ) following presentation of the sad facial expressions (F = 4.5, *p* < 0.03) as well as in response to the neutral facial expressions (F = 4.5, *p* < 0.03). A history of adolescent extreme binge drinking also was associated with decreases in connectivity (Figure 4B) in all three cortical areas (FZ: F = 6.3, *p* < 0.01; CZ: F = 5.4, *p* < 0.02; PZ: F = 6.1, *p* < 0.01) in response to sad facial expressions, as indexed by local phase locking within the cortical areas over time. Additionally, reduction in local phase locking in those with an adolescent history of extreme binge drinking was observed in response to the happy facial expressions in the central (CZ: F = 5.6, *p* < 0.018) and parietal (PZ: F = 5.0, *p* < 0.026) cortical areas. 

The percent of correct identifications of the three facial expressions was compared between those participants with an adolescent history of extreme binge drinking and those without. A decrease in the percentage of correctly identified sad faces (no binge drinking: 90%, extreme binge drinking: 86%, F = 11.8, *p* < 0.001) and neutral faces (no binge drinking: 96%, extreme binge drinking: 93%, F = 12.1, *p* < 0.001) showed significant declines among those with a history of extreme binge drinking, but no significant differences between groups were found for the percent correctly identified happy faces.

**Figure 5:**
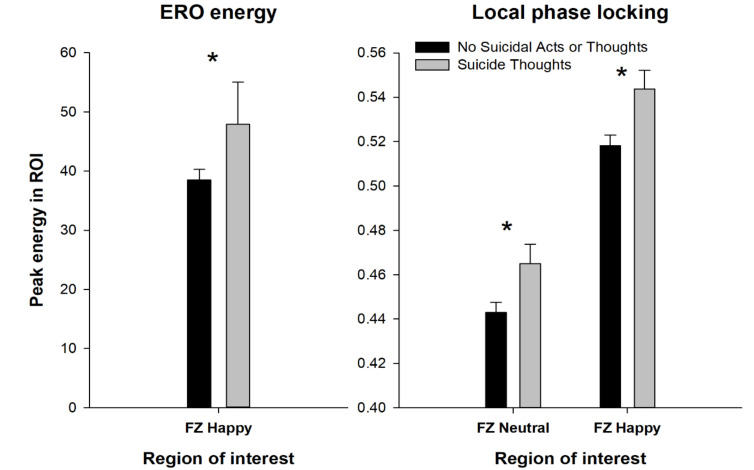
Subjects with no suicidal activity compared to those with suicidal thoughts by their (ERO) energy and connectivity shown in the delta frequency band (1–4 Hz) for the 200 to 500 ms time interval. * indicates ANOVA co-varied for age and race *p*-value less than 0.05.

Further, suicidal thoughts and acts were associated with significant differences in ERO energy and connectivity, as seen in Figure 5. History of suicidal thoughts (without suicidal acts) was associated with increases in delta ERO energy in response to happy faces in the frontal cortex (F = 4.3, *p* < 0.038), as well as with increases in connectivity, as indexed by local phase locking over time, to neutral (F = 4.2, *p* < 0.04) and happy (F = 5.35, *p* < 0.02) facial expressions in the frontal cortex when compared with no suicidal activity. The percent of correct identifications of the three facial expressions for those participants with a history of suicidal thoughts and those without showed an increase in the percentage of correctly identified sad faces (suicidal thoughts: 90.5%, no suicidal thoughts: 88.7%, F = 3.9, *p* < 0.05) in participants with suicidal thoughts, but no significant differences between groups were found for the percent correctly identified neutral or happy faces.

**Figure 6:**
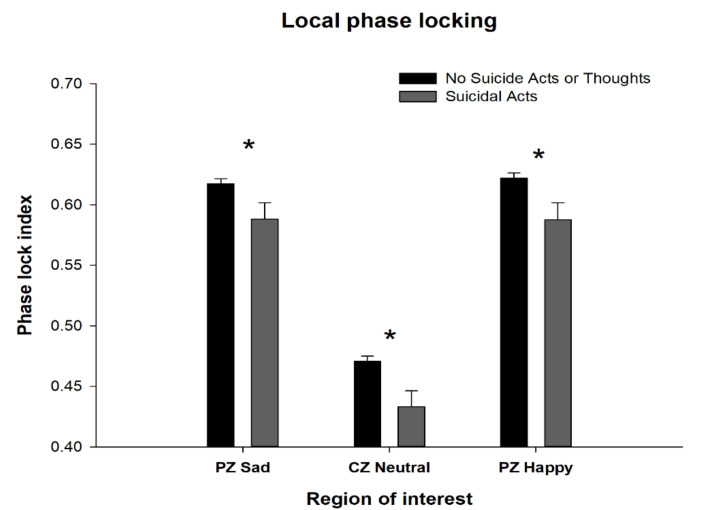
Subjects with no suicidal activity compared to those with suicidal acts by their (ERO) connectivity (local phase locking) shown in the delta frequency band (1–4 Hz) for the 200 to 500 ms time interval. * indicates ANOVA co-varied for age and race *p*-value less than 0.05.

Suicidal acts, as compared to no suicidal acts or thoughts history, were found to be associated with a different pattern of ERO changes. A decrease in ERO local phase locking was seen in the parietal cortex in response to sad faces (F = 4.5, *p* < 0.03), in the central cortex (F = 4.5, *p* < 0.035) to neutral faces, and in the parietal cortex to happy faces (F = 6.2, *p* < 0.01), as seen in Figure 6. The percent of correct identifications of the three facial expressions for participants with a history of suicidal acts compared to those without a history of either acts or thoughts showed a decrease in the percentage of correctly identified neutral faces (suicidal acts: 93.5%, no suicidal acts: 95.7%, F = 8.2, *p* < 0.004), with no significant differences between groups for the percent correctly identified sad or happy faces.

## 4. Discussion

Recent data suggest the prevalence of binge drinking, as defined by consuming ≥5 drinks per occasion, has been in an overall decline in the U.S. [108]. However, many adolescents and young adults still engage in harmful drinking and report drinking in amounts that far exceed the traditional binge threshold, consuming more than 10 or even 15 drinks per occasion [6,7,8,9]. Among high school seniors who participated in the Monitoring the Future (MTF) study between 2005 and 2011, 10% reported 10+ and 5.6% reported 15+ drinks per occasion in the past 2 weeks [6]. The MTF study did report some drinking data based on self-identified race, but they did not include data for American Indian (AI) or Asian participants or for Hispanic subgroups [6]. In the current study sample of AI and Mexican American (MA) young adults, 20% reported extreme binge drinking (mean drinks per occasion = 17) during adolescence. Extreme binge drinking was more common in AI (compared to MA), males, those of younger age, those with less education, unmarried participants, and those from lower socioeconomic groups. Such demographic differences between extreme binge drinkers and those that did not engage in extreme binge drinking are consistent with MTF study findings, where binge drinking was found to be more common in males, those who self-identified as white, those with parents who had no college education, and those who lived in the Midwest and/or rural areas [6]. However, our study observed higher rates of extreme binge drinking in AI and MA participants—a finding consistent with reports showing high drinking rates in AI teens and young adults (see [15,109,110,111,112]). Our findings also support studies showing that more Hispanic adolescents (i.e., youth between ages 12–17) report engaging in binge drinking during the past month relative to other ethnic minority youth including Native Hawaiians or other Pacific Islanders, Black, and Asian adolescents [113]. 

Binge drinking during adolescence has been associated with an increased risk for AUD in young adulthood [10], as well as other mental health risks [11], including increased risk for suicide [12,13,14]. Suicidal behaviors range in severity from suicidal ideation, suicide plans to suicide attempts to death by suicide. In the present study, a history of extreme binge drinking was associated with a significantly increased risk of suicidal acts (suicide attempts, death) but not suicidal thoughts (suicidal ideation, plans). These data suggest that alcohol more specifically moves people from ideation to acts rather than increasing ideation in those with no suicidal thoughts. A history of suicidal thoughts was found to be more common among MA compared to AI participants, which builds on previous studies that show, even when adjusting for a wide variety of demographic covariates [114], that Mexican American middle school students were 1.8 times more likely to have high suicidal ideation than their European American classmates. We found suicidal acts were more common in AI compared to MA participants, and known suicide mortality was observed among AI participants alone. These data also align with recent findings indicating that AI/AN youth and young adults show suicide mortality rates that are more than 2 to 3 times higher than their same-aged European American, African American, and Asian/Pacific Islander peers [115]. Such findings spotlight the need for prevention and intervention efforts in this high-risk group [116] and underscore the urgency for increased research within adolescence specifically. These data also align with findings showing increased risk of suicide among AI/ANs in association with alcohol intoxication [117]. 

In addition to providing descriptive data on extreme binge drinking and suicide risk, a key goal of the present analyses was to identify ERO signatures in the delta frequency range among MA and AI participants and test for associations with extreme binge drinking during adolescence and with suicide risk. This study found increases in energy and decreases in phase locking of delta oscillations in response to the target (happy and sad facial expressions) as compared to non-target (neutral expressions) stimuli, especially in the posterior leads. Increased energy in delta EROs in posterior leads in response to facial stimuli has been reported previously [69,71,72,118,119], suggesting the importance of these sensitive response patterns as emotion-based measures of alcohol-related pathology. Participants in this sample with a history of adolescent extreme binge drinking demonstrated increases in delta ERO energy in the parietal lead following presentation of sad and neutral facial expressions as compared to those without an adolescent history of extreme binge drinking. A history of adolescent extreme binge drinking was also associated with decreases in connectivity in all three cortical areas in response to sad facial expressions, as indexed by local phase locking. There have been a few studies that have evaluated ERO energy in specific frequency bands in individuals with alcohol-associated pathology (see [36,38,39,55,56,58,59,120]. In two sets of studies, it was demonstrated that both alcoholics [66,120] and young offspring of alcoholics at high risk for alcohol use disorders [67], show decreases in delta oscillations to target stimuli during a visual oddball task. Thus, responses to emotional facial stimuli may elicit different responses from those at risk for AUD than other visual tasks may do. 

In contrast to electrophysiological studies [121,122], there have been numerous studies that have evaluated behavioral measures of facial recognition in alcohol-related pathology. Although most studies used relatively small numbers of participants [123,124,125,126,127,128,129,130], a systematic review found trends for impairments by alcoholics in facial recognition tasks [131], and a meta-analysis of facial emotional recognition found deficits in individuals with AUDs with an effect size of 0.6 [132]. The recognition of facial emotion has also been evaluated in adolescents, where initiation of substance use was associated with enhanced recognition of angry emotions [133]. Altered emotional processing has also been documented in adolescent binge drinkers [134,135,136]. Our findings support previous investigations demonstrating recognition deficits in at-risk adolescents. However, we specifically found a decrease in the percentage of correctly identified sad and neutral faces, and not happy faces, in those with a history of adolescent binge drinking, suggesting an affective bias in recognition. 

Differences in the processing of emotional expressions in participants with suicidal thoughts and acts also has been reported previously. An increased tendency to interpret neutral facial expressions as sad has been reported in adult patients with “affective temperaments” in one study [137], and more errors in the recognition of facial expressions of “disgust” in another study [138]. Youth with a history of engaging in non-suicidal self-injury and those with past suicide attempts were found to make more errors in sad face recognition [139], and children with suicidal ideation were found to misclassify angry emotions as sad [140]. The percent of correct identifications of the three facial expressions were compared between participants with a history of suicidal thoughts and those without in the present study. An increase in the percentage of correctly identified sad faces was seen in those with suicidal thoughts, whereas a decrease in the percentage of correctly identified neutral faces was found in those with more severe suicidal behaviors, including a suicide attempt history and death by suicide. Taken together, these studies suggest an emotional bias towards misclassifying or incorrectly identifying sad or neutral facial expressions in association with suicidal behaviors. 

Electrophysiological responses to emotional stimuli also have been investigated in participants with suicidal thoughts and acts. Albanese et al. [141] used a go/no-go task to distinguish participants with suicidal ideation from those who had made a suicide attempt. They found participants with a history of suicide attempts exhibited deficits in detecting “the need for inhibitory control,” as indexed by a more positive change in the N2 response of the ERP. Larger P3a components in a passively-presented auditory ERP paradigm were found in another sample of adolescent inpatients with suicidal behavior [142]. Activated dorsolateral prefrontal cortex has been demonstrated in fMRI studies of youth that attempted to regulate their emotional responses to negative pictures [143] as well. In the present study, suicidal thoughts and acts were associated with significant differences in ERO energy and connectivity in the delta frequency range. A history of suicidal ideation without suicidal acts was associated with increases in delta ERO energy and phase locking in response to happy faces in the frontal cortex. These findings build upon past reports and suggest that enhanced fMRI, ERO and ERP energy may represent potential biomarkers of suicidal risk. 

In the present sample of young adults, similar to findings for adolescent binge drinking, suicidal acts (as compared to no suicidal acts or ideation) were associated with a decrease in ERO local phase locking in the parietal cortex in response to sad and happy faces and in the central cortex in response to neutral faces. There have been no studies to date that have evaluated ERO connectivity, as indexed by local phase locking, in relation to adolescent binge drinking and/or suicidal behaviors. However, there have been some fMRI imaging studies that indexed connectivity in samples of binge drinkers as well as in studies of suicide attempters. Decreases in right amygdala-orbital frontal cortex functional connectivity has been reported in adult binge drinkers [144]. A decrease in fMRI connectivity between prefrontal cortex and striatal regions have also been shown in adult rats that were treated with “binge” amounts of alcohol during adolescence [145]. Measures of fractional anisotropy (FA) in accumbo-frontal brain circuits, an index of white matter microstructure and an anatomical measure of connectivity, has been shown to be lower in adolescents who began binge drinking sooner [146]. In that study, the authors hypothesized that lower FA may suggest delayed maturation of prefrontal white matter that could be associated with “less top-down control over striatal sensitivity to reward” [146]. Additionally, fMRI measures of connectivity have been used to distinguish participants with suicidal thoughts and behaviors. Elevated activity and reduced anterior cingulate gyral-insula functional connectivity have been shown in response to angry faces in adolescents with a suicide attempt history [147]. In another study, youth with a history of suicide attempts were found to have lower grey matter volume in orbitofrontal cortex, lower white matter integrity, and decreased functional connectivity between amygdala and PFC when viewing emotional faces [148]. These authors suggested that decreased functional and structural connectivity in a ventral fronto-limbic neural system subserving emotional regulation may be a measure of the severity of suicidal ideation and attempt lethality [148].

The neural mechanisms that may underlie the changes seen in ERO delta activity in participants in the present study with extreme binge drinking and/or suicidal behaviors remains unknown. However, there are several preclinical studies that have demonstrated that binge-like alcohol administration, to adolescent rodents, using multiple routes of alcohol administration and rodent strains, results in persistent decreases choline acetyltransferase immunoreactivity (ChAT+IR) in forebrain cholinergic neurons [149,150,151]. The 20 to 30% decrease in ChAT+IR by AIE is also associated with loss of muscarinic and nicotinic cholinergic receptor mRNA [149]. These findings may be important to the understanding of the current results as in another set of studies, we have demonstrated that lesions to the basal forebrain cholinergic neurons, in rats, results in increases in delta ERO energy [152]. Thus, it is theoretically possible that the increase in delta ERO energy seen in the present study in extreme binge drinkers may be the result of changes in cholinergic tone.

## 5. Conclusions

We found extreme binge drinking was common (20%) in this population of AI/MA young adults, and it was associated with a significantly increased risk for more severe suicidal behaviors, including suicide attempts and death by suicide, but not suicidal thoughts. Increased energy and phase locking of delta oscillations were evident in response to the target stimuli (happy and sad facial expressions) as compared to non-targets (neutral expressions), especially in the posterior leads, which validated this paradigm for future research with young adult samples. Participants with a history of adolescent extreme binge drinking demonstrated increases in delta ERO energy in the parietal lead following presentation of the sad and neutral facial expressions and compared to those without an adolescent history of extreme binge drinking. A history of adolescent extreme binge drinking was also associated with decreases in connectivity in all three cortical areas in response to sad facial expressions, as indexed by local phase locking. A decrease in the percentage of correctly identified sad and neutral faces, but not happy faces, was also found in those with a history of adolescent binge drinking, suggesting an affective bias in recognition. Finally, suicidal thoughts and acts were associated with significant differences in ERO energy and connectivity in the delta frequency range as well. Suicidal ideation, without suicidal acts, was associated with increases in delta ERO energy and phase locking in response to happy faces in the frontal cortex, whereas more severe suicidal acts, as compared to no suicidal acts or ideation, predicted a decrease in ERO local phase locking in the parietal cortex in response to sad and happy faces and in the central cortex in response to neutral faces. These studies suggest that ERO energy and phase locking may represent a putative biomarker of adolescent extreme binge drinking and suicide risk. 

The results of this study should be interpreted in the context of several limitations. First, the findings may not generalize to other Native American or Mexican American young adults, especially since the sample obtained was a community participation sample and not a randomized sample. Second, comparisons to non-Native American or non-Mexican populations may be limited by differences in a host of potential genetic and environmental variables. Next, some of the data collected were retrospective and are subject to recall bias. The specificity of the reported findings need to be tested in further analyses, for instance the ERO changes reported could also be a reflection of familial risk for AUD. Thus, a longitudinal study to explore temporal relationships between family history, binge drinking and variables collected in this study would represent a more powerful study design and may inform important developmental pathways of risk. Additionally, it may be that extreme binge drinking during sensitive developmental periods during adolescence results in brain changes that predispose someone to suicidal behaviors. The study also did not completely control for multiple comparisons. The ERP paradigm used in this paper may confound emotional recognition and processing with target detection. Despite these limitations, our findings represent an important step in an ongoing investigation to understand developmental determinants associated with substance use disorders in these high-risk, understudied ethnic groups to inform early detection and treatment.

## Figures and Tables

**Table 1:** Demographic characteristics of adolescent binge drinking and suicidal activity in Mexican American (*n* = 705) and American Indian (*n* = 479) participants.

Demographic Characteristic	No Suicidal Activity	Suicidal Thoughts	Suicidal Acts	No Adolescent Binge	Adolescent Binge	Extreme Adolescent Binge	Overall (*n* = 1184 ^1^)
	n (%)	n (%)	n (%)	n (%)	n (%)	n (%)	n (%)
Race		*			*	*	
American Indian	330 (27.9)	86 (7.3)	63 (5.3)	152 (13.1)	136 (11.7)	180 (15.5)	479 (40.5)
Mexican American	469 (39.6)	177 (14.9)	59 (5.0)	440 (37.8)	200 (17.2)	55 (4.7)	705 (59.5)
Gender			*			*	
Male	353 (29.8)	116 (9.8)	38 (3.2)	238 (20.5)	136 (11.7)	131 (11.3)	507 (42.8)
Female	446 (37.7)	147 (12.4)	84 (7.1)	354 (30.4)	200 (17.2)	104 (8.9)	677 (57.2)
Married							
Yes	96 (8.1)	26 (2.2)	10 (0.8)	79 (6.8)	35 (3.0)	17 (1.5)	132 (11.1)
No	703 (59.4)	237 (20.0)	112 (9.5)	513 (44.1)	301 (25.9)	218 (18.7)	1052 (88.9)
Employed						*	
Yes	392 (33.7)	131 (11.3)	59 (5.1)	331 (28.9)	163 (14.2)	77 (6.7)	582 (50.0)
No	393 (33.8)	130 (11.2)	59 (5.1)	256 (22.4)	165 (14.4)	152 (13.3)	582 (50.0)
Income ≥ $20,000/year						*	
Yes	521 (48.6)	178 (16.6)	72 (6.7)	408 (38.6)	237 (22.4)	119 (11.3)	771 (71.9)
No	199 (18.5)	64 (6.0)	39 (3.6)	136 (12.9)	69 (6.5)	87 (8.2)	302 (28.1)
	Mean (SD)	Mean (SD)	Mean (SD)	Mean (SD)	Mean (SD)	Mean (SD)	Mean (SD)
Age (years)	22.86 ± 4.0	22.89 ± 3. 9	22.63 ± 3.6	23.14 ± 3.9	22.78 ± 4.0	22.2 ± 3.9 *	22.84 ± 3.8
Education (years)	12.71 ± 2.0	12.91 ± 2.0	12.11 ± 1.8 *	13.14 ± 1.9	12.74 ± 1.7 *	11.55 ± 1.5 *	12.69 ± 1.7

^1^ 21 subjects had missing adolescent drinking data (*n* = 1163). * indicated *p* < 0.01 compared to control group.
